# *Notes from the Field*: Tuberculosis Control Activities After Hurricane Harvey — Texas, 2017

**DOI:** 10.15585/mmwr.mm6649a5

**Published:** 2017-12-15

**Authors:** Sandra Morris, Mark Miner, Tomas Rodriguez, Richard Stancil, Dana Wiltz-Beckham, Terence Chorba

**Affiliations:** ^1^Texas Department of State Health Services; ^2^Division of Tuberculosis Elimination, CDC; ^3^Houston Health Department, Texas; ^4^Harris County Health Department, Texas.

On September 14, 2017, the Texas Department of State Health Services (DSHS) reported that Hurricane Harvey had caused 82 deaths in Texas during August 25–August 30, 2017 (*1*), with property damage that could total $180 billion (*2*). Houston alone received 45 inches of rain from August 24 to September 1, 2017, and some parts of Texas received 60 inches or more. Dozens of inches of rain also fell on the cities of Port Arthur and Beaumont. Several local health departments experienced closures during the week of August 28 and resumed operations the week of September 5 under emergency conditions.

The Texas DSHS uses federal and state funding for tuberculosis (TB) surveillance, prevention, and control activities in eight DSHS health service regions, 31 local health departments, and four binational TB projects. In advance of major storms, TB programs have activated established protocols for providing patients with medications to take on their own, and for providing contact information to give to health departments in case patients become displaced. Line listings of patients are closely monitored to account for all patients after the storm, and treatment duration is frequently extended to allow for medication doses that were missed. Information exchange among neighboring local, regional, or state programs is often necessary.

Directly observed therapy (DOT) of patients taking each dose of their TB medications is a cornerstone of TB control activity, and video-enabled DOT using electronic devices, such as smart phones, has become a useful tool for patients who cannot visit, or be visited by, a health care provider (*6*). Lessons learned regarding management and follow-up of TB patients on treatment during Hurricane Katrina in 2005 (*3*) were applied during hurricanes Gustav and Ike in 2008 (*4*), Sandy in 2012 (*5*), and Harvey in 2017. Whereas approximately half of the TB patients in New Orleans, Louisiana, fled the state during Hurricane Katrina (*3*), TB patients in Texas during Hurricane Harvey typically remained close to their usual residence (at home, with relatives, or in shelters).

Immediately after Hurricane Harvey, the DSHS TB program directly contacted all affected regional and local health departments to determine the status of high-priority TB patients (persons with new TB diagnoses, infectious patients, and children), and relayed status of patient care, health care worker safety, and needs of local and regional health departments to CDC. In addition, surveillance questionnaires were distributed to temporary shelters to identify residents or volunteers exhibiting signs and symptoms of TB. Although TB control personnel in Texas were personally affected by the storm’s damage, they remained on duty, with some staff members traveling into flooded communities to follow up patients. 

A total of 282 TB cases from 17 affected local or regional health departments, including 212 (75%) from one large urban county, were high priority TB cases with confirmed disease. Response efforts by affected local and regional health departments ensured that all but two of the 282 persons were accounted for within a week after the storm began. The remaining two were located the following week and connected to care. Sixty-one patients had already been placed on video-enabled DOT, 30 had TB disease (cases), and 31 had latent TB infection and needed DOT. Fifty-nine (97%) were monitored successfully and did not miss any medication doses. The aforementioned two patients who were lost during the storm and found a week later had TB disease (cases). Although respiratory illnesses among shelter residents were reported, no suspected cases of undiagnosed TB disease were identified. 

Each year, the upcoming hurricane season provides opportunities to develop, test, and implement preparedness plans for continuity of patient care. During Hurricane Harvey, the high proportion of patients successfully managed through video-enabled DOT demonstrates that video-enabled DOT can help ensure TB treatment completion when regular treatment options have been disrupted by a major storm or other disasters.
